# The COVID-19 Pandemic and Economic Growth: Theory and Simulation

**DOI:** 10.3389/fpubh.2021.741525

**Published:** 2021-09-17

**Authors:** Lijin Xiang, Mingli Tang, Zhichao Yin, Mengmeng Zheng, Shuang Lu

**Affiliations:** School of Finance, Shandong University of Finance and Economics, Jinan, China

**Keywords:** COVID-19 pandemic, economic growth, health capital, public health, interdisciplinary analysis

## Abstract

The outbreak of COVID-19 pandemic has caused profound consequences on world economy. In order to explore the long-term impact of the pandemic on economic growth and the effects of different policy responses, this paper combines economic theory with epidemiological model to construct an interdisciplinary model, in which labor supply is dynamically constrained by pandemic conditions. Analysis of model equilibrium suggests that outbreaks of infectious disease reduce labor supply and negatively affect economic output. The accumulation of health capital can suppress the spread of disease and improve the recovery rate of infected individuals, which will alleviate the labor supply constraint caused by the pandemic and lead to an increase in output and consumption. The model is then calibrated to Chinese economy. The simulation results imply that government's public health policy can enhance the role of health capital in promoting economic growth. But the marginal effect of certain policies is diminishing. Therefore, the government needs to balance pandemic prevention and control costs and marginal benefits when formulating public health policies. When the pandemic is under control, the resumption of production is feasible and the economic stimulus package could lead to economic recovery.

## Introduction

In recent years, outbreaks of infectious diseases, such as severe acute respiratory syndrome (SARS) and Middle East respiratory syndrome (MERS), bring severe negative shock to the world economy. And, the cross-border trade and travel have facilitated the international spread of the pathogens (1).

By the end of 2019, several cases of COVID-19 have been identified in China. A total of 830 cases were confirmed between the discovery of the virus in early 2020 and the “Lockdown” of Wuhan city on 23 January 2020. The Chinese government has extended the Chinese New Year holiday to reducing population mobility across regions. And social distancing and community quarantine procedures are carried out to slow down the spread of the virus. By 16 February 2020, more than 70,000 cases had been confirmed nationwide, while the number of new cases continued to fall and the number of cured cases rose sharply. After hard fighting against the pandemic, Wuhan City reopens at 8 April. Since then, only occasional imported infections have been detected and properly handled in a couple of regions. And there is no second wave of tremendous outbreak in China. On 23 May 2021, a newly-discovered COVID-19 patient in Guangzhou was confirmed to be infected with the “delta variant” of the novel coronavirus. Fortunately, under regional lockdown and strict epidemic control measures, regional spread was quickly blocked.

These facts reflect the importance of timely and effective infectious disease prevention and intervention measures by the government. However, the prevention and control of infectious diseases requires limiting resident mobility and aggregation and reducing social and economic activities, that significantly suppress both the demand and supply of the economy (35). At the same time, the fight against the pandemic delays the resumption of production in various industries and causes additional prevention costs, which greatly affects economic output. So, it is crucial for the authorities to balance the efforts to control the epidemic and the resumption of economic activity.

The main contribution of this paper to the existing literature lies in the following aspects: (i) In the theoretical model, through the endogenous health capital accumulation, the spread of infectious diseases is linked to the dynamics of labor supply in the economy, so as to realize the interdisciplinary integration of economic theory and infectious disease dynamics model; (ii) After+ deriving the differential equation of uninfected population from the infectious disease model, it is introduced into the central planner's optimization problem as a dynamic constraint for labor. The interdisciplinary theoretical framework makes it possible to obtain the steady-state of both endogenous economic variables and health capital and to ravel the dynamic nexus of COVID-19 pandemic and the macroeconomic fluctuation; and (iii) The simulation studies based on the calibrated model shed light on policymakers' trade-off between pandemic prevention and economic development. From a social-economic perspective, we discuss the effects of public health policy implementation and economic stimulus under different conditions.

The following sections of this paper are organized as follows: section Literature Review reviews existing literatures. Section Theoretical Model develops an interdisciplinary theoretical framework to explore multiple equilibria that may exist in the economic system during different stages of pandemic. The fourth section calibrates the model and perform steady-state analysis. The fifth section includes analysis of the policy response and the effects. And the last section concludes the paper.

## Literature Review

We review related studies from following aspects: (i) the empirical evidence of economic consequences after the hit of pandemic; (ii) the pandemic induced poverty trap; (iii) the mechanism that how pandemic affects the economic growth; and (iv) the policy response.

### Pandemic and Economic Growth

The economies of many low-income countries have been hit hard by infectious diseases. Gallup and Sachs (2) analyze the relationship between poverty and malaria for tropical and subtropical countries, and find that the areas with severe malaria have been in poverty for a long time and accompanied by anemic economic growth. For developed countries, Holtkamp et al. (3) assessed the impact of the swine fever epidemic on the US economy from 2005 to 2010 using data on agricultural enterprises disclosed by the US Department of Agriculture. The results showed that the average annual economic loss caused by the epidemic was about 664 million US dollars. Joo et al. (4) document a significant negative impact on South Korea's overall economic growth. Zhang et al. (5) illustrate that pandemic leads to unpredictable socioeconomic and long-term effects in low- and middle-income countries, and people or countries with lower socioeconomic status are worse off in these situations. Brown et al. (6) state that the COVID-19 pandemic has had a profound impact on American lives. In the months since the COVID-19 outbreak was first diagnosed, it has spread to more than 200 countries and all U.S. states. The pandemic has had a negative impact on global economic growth for more than nearly a century. Estimates to date suggest that the virus reduces global economic growth to an annualized rate of −4.5 to −6.0% in 2020.

### Pandemic Induced Poverty Trap

The infectious diseases will weaken or even deprive the infected person's ability to work, which leads to lower income level and labor supply. As a result, there would be lack of investment and other factor input, which eventually suppresses economic growth. In addition, the impact of infectious diseases and related pandemic prevention measures on human capital accumulation will inhibit the improvement of production efficiency and weaken the momentum of economic growth.

First of all, infectious diseases directly lead to a decline in the income of infected persons, creating a poverty trap. For hundreds of years, the economies of most countries in the world have achieved rapid economic growth, but more than one-sixth of the world's population is still in poverty for a long time. This widespread and persistent poverty is closely related to infectious diseases [7, Gallup and Sachs, (2)]. A large number of studies have shown that extreme poverty is often accompanied by infectious diseases in areas where poverty induces the occurrence and spread of diseases, and disease-driven poverty traps further restrict economic development (8, 9). The reason for the formation of the poverty trap is that low-income people will quickly lose their means of production due to infectious diseases. After the epidemic is over, the lack of labor tools and labor capital has aggravated the persistence of poverty (10). Bonds et al. (11) and Ngonghala (12) integrated economic analysis on the basis of infectious disease models and found that in the poverty trap, initial economic, and epidemiological conditions determine the health of a society and the long-term trajectory of economic growth. The government's commitment to improving the health of the population is a necessary condition for getting out of poverty. Shen et al. (13) provide evidence that links rise in regional income inequality in China to the outbreak of COVID-19.

### The Mechanism

The poverty trap caused by infectious diseases may cause economic growth to stagnate in the long run. Chakraborty et al. (14) believe that the poverty trap caused by infectious diseases will change the investment tendency of economic entities. At the same time, disease will directly lead to a decline in labor supply, and the input of factors in the economy will be significantly negatively affected. Therefore, in the long run, infectious diseases may cause a “growth trap”: the economy deviates from the original equilibrium growth path, and growth slows or even stagnates.

Infectious diseases weaken the accumulation of human capital and restrict the improvement of productivity. The impact of the spread of the virus on human health will eventually be transmitted to the economic production process through human capital channels (15, 16). In the long run, the continuous spread and repeated outbreaks of infectious diseases will also reduce the life expectancy of the population, thereby further reducing the accumulation of human capital in society. This phenomenon is very common in low-income countries where AIDS and malaria are endemic (17, 18). Fortson (19) studied the impact of the AIDS epidemic on human capital investment and economic growth in Africa. He found that areas with high HIV infection rates have a relatively large decline in education and a decline in human capital investment. The slow accumulation of human capital has dragged down the African economy. The theoretical research of Goenka and Liu (20) based on the endogenous growth model including human capital pointed out that there are multiple balanced growth paths for economic growth under the epidemic of infectious diseases. The restriction of infectious diseases on the accumulation of human capital is an important reason why many underdeveloped countries are unable to achieve economic growth. And the financial markets are also subject to the COVID-19 pandemic (21).

### Policy Response

Zhang et al. (5) concluded from a review of the response to the COVID-19 pandemic in China and some other middle-income countries that the most important aspect of responding to an infectious disease outbreak is the preparedness and response of hospitals as the first line of defense against the pandemic. They argue that as the influenza season progresses, hospitals use the experience and lessons learned in the early stages of the pandemic to continually adapt their response to effectively deal with potential public health emergencies. And as Pardhan and Drydakis (22) point out that GDP per capita is associated with a lower rate of new COVID-19 cases, thus the economic performance should be a critical health priority for the policy makers.

Using combined the data of “SARS” cases with the epidemiological parameters of the COVID-19 pandemic, Yang et al. (23) adopt the method of recurrent neural network to carry out SEIR model estimation in different scenarios. They find that the government's swift intervention against the spread of the virus played a significant role in controlling the scale of infection. Goenka and Liu (24) analyzed the endogenous fluctuations of macro variables caused by infectious diseases in a growth model, and explored how to stabilize the macro economy through countermeasures such as vaccines or isolation. They argue that even when the virus cannot be completely eliminated, the prevention and control measures against the spread of the disease can smooth economic fluctuations and bring welfare gains.

Bogoch (25) use flight data from Guinea, Liberia, and Sierra Leone to study and found that thanks to timely flight control by the government, the number of passengers potentially infected with Ebola virus in the three countries decreased by 51, 66, and 85%, respectively. According to the counterfactual analyses by Qiu et al. (26), more than 1.4 million cases and 56,000 deaths may have been avoided as a result of the national and provincial public health measures imposed in late January in China.

In summary, existing studies have explored the negative impact of the pandemic on economy and the government's response measures. This paper introduces dynamic labor supply and time varying health capital accumulation in a broad sense into the canonical growth model. Our approach models the constraints imposed to the labor supply by the pandemic, and dynamically links government intervention, health capital, and disease transmission. We have developed an interdisciplinary theoretical model and discuss in depth the pandemic induced economic fluctuation and the government's response.

## Theoretical Model

Almond (27) have pointed out that infectious diseases will directly increase the health costs of residents, hinder the accumulation of human capital, reduce labor time, and ultimately have a negative impact on economic growth in the long-term. Therefore, using the variable of labor supply as an intermediary can link the dynamic spread of infectious disease with the fluctuation of macroeconomy. Thus, it is able to carry out economic analysis under a pandemic shock [24, Augier and Yaly, (36)].

**Figure 1:**
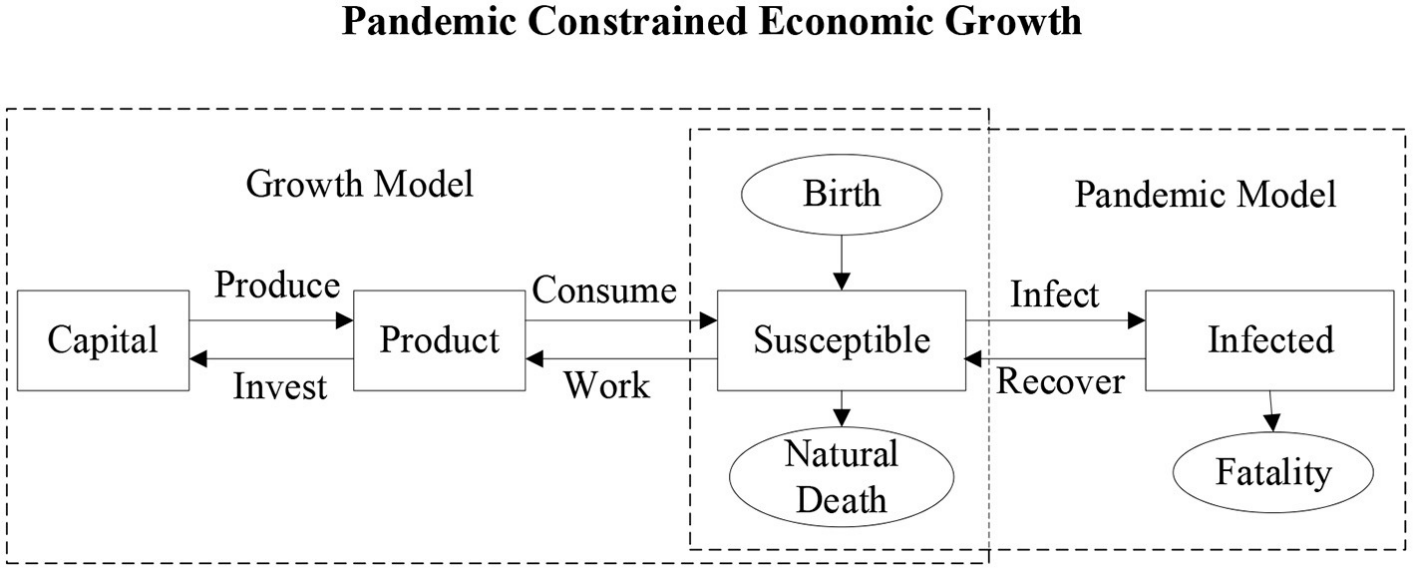
Interdisciplinary theoretical model.

This article starts from the perspective of infectious disease dynamics, and theoretically explores the differential equations that must be satisfied by the dynamic change of the labor force scale (healthy population) in the presence of an epidemic. At the same time, from an economic perspective, the most core supply and demand variables are closely related to labor. Residents provide labor for manufacturers to obtain income, and then allocate income to consumption and investment. Manufacturers use the labor provided by residents and the capital generated by investment accumulation to carry out product production activities. Therefore, through labor supply, a core macroeconomic variable, the impact of infectious diseases can be introduced into the economic model. At the same time, macroeconomic changes will change household income, affect the accumulation of health capital, and in turn change the spread of infectious diseases. The diagram of the interdisciplinary theoretical model is shown in Figure 1.

### The Dynamic Spread of Infectious Diseases

The literature on mathematical modeling of the dynamic process of spread of infectious diseases can be traced back to Kermack and McKendrick (28). At present, the most widely used classical theoretical framework in the field of infectious diseases is the Susceptible-Infective-Susceptible (SIS) model (29, 30). Different infectious disease dynamics models have certain differences in the settings. In the SIS model, the infected person will be cured (or self-healed) at a certain rate and reclassified into the susceptible population. For diseases that can obtain immunity after cure, according to the duration of immunity, they can be divided into two types: Susceptible-Infective-Recovered (SIR) and Susceptible-Infective-Recovered-Susceptible (SIRS). Among them, the situation where the cured population obtains permanent immunity applies the SIR model, and the situation where the cured population only obtains temporary immunity applies the SIRS model.

In order to ensure that the results will not underestimate the negative impact of the epidemic on the macro economy, we use the SIS model framework to characterize the dynamic spread of the novel coronavirus. The model assumes that the cured infected cases can be re-infected^1^. And in such settings, the threat of rapid virus mutation and the emerge of new variants will not be overlooked. Goenka and Liu (24) simplify the SIS model into a two-equation differential equation system under the additional assumption that the total population size is constant. They explore the endogenous fluctuations of macro variables caused by infectious diseases in a growth model. However, the assumption of population size is too strict to match economic reality. This paper follows the basic hypothesis of Gersovitz and Hammer (30) and believes that the population size is continuously increasing, which means that the birth rate is greater than the death rate.

We denote the total population during the period *t* as *N*_*t*_, which includes the susceptible population who are not infected by the virus*N*_*t*_, and the infected population *I*_*t*_^2^. Thus, the total population can be expressed as the sum of the numbers of two types of people:


(1) $$\begin{array}{l} N_{t}=S_{t}+I_{t} \end{array}$$

The law of motion for the total population is characterized by Equation (2):


(2) $$\begin{array}{l} N_{t}=bN_{t}-dN_{t} \end{array}$$

where, the birth rate and death rate are denoted as *b* and *d*, respectively.

Susceptible population will become infected because of contact with infected cases. Part of infected population will become susceptible again because of self-healing or being cured. Such a dynamic relationship can be expressed by the following differential equations:


(3) $$\begin{array}{l} {Ṡ}_{t}=bN_{t}+\gamma I_{t}-\alpha \frac{I_{t}}{N_{t}}S_{t}-dS_{t} \end{array}$$

(4) $$\begin{array}{l} {İ}_{t}=\alpha \frac{I_{t}}{N_{t}}S_{t}-dI_{t}-\gamma I_{t} \end{array}$$

Among them, the death rate of the infected person is set as *d*, the recovery rate is γ, and the contact rate between individuals is α. Under the assumption of random contact, the probability of susceptible people contact with infected cases is the proportion of infected people in the total population: *i*_*t*_ = *I*_*t*_/*N*_*t*_. Naturally, the susceptible population accounts for the proportion of the total population is *s*_*t*_ = *S*_*t*_/*N*_*t*_ = 1 − *i*_*t*_. The death rate of infected cases is a redundant parameter for the above-mentioned dynamic system. For the sake of simplicity, it is set to be the same as the death rate of the whole population, which will not affect the subsequent analysis^3^.

By further simplifying the Equations (2, 3, 4), we can get:


(5) $$\begin{array}{l} {ṡ}_{t}=i_{t}\left(b+\gamma -\alpha s_{t}\right)=\left(1-s_{t}\right)\left(b+\gamma -\alpha s_{t}\right) \end{array}$$

It can be found that the change in the proportion of susceptible population is closely related to the birth rate, disease recovery rate, and the contact rate. Under the impact of the pandemic, the susceptible population constitute as the real labor supply in economic activities, not the total population. Therefore, Equation (5) can portrait the dynamic changes of labor supply in the economy when the pandemic is raging.

### Government Intervention, Health Capital Accumulation, and Labor Supply

In this theoretical model, lowercase letters are used to represent the per capita level of each economic variable. According to the previous analysis, labor supply comes from uninfected population, formally *L*_*t*_ = *S*_*t*_. Thus, according to Equation (5), we have:


(6) $$\begin{array}{l} {\dot{l}}_{t}=\left(1-l_{t}\right)\left(b+\gamma -\alpha l_{t}\right) \end{array}$$

Where, *l*_*t*_ = *L*_*t*_/*N*_*t*_ not only represents the proportion of uninfected people in the population, but also per capita labor supply.

Representative firm uses the physical capital and labor from representative household to produce products. The production function is:


(7) $$\begin{array}{l} y_{t}=ak_{t}^{\eta }l_{t}^{1-\eta } \end{array}$$

The final product produced by the manufacturer is consumed by the household or invested in physical capital and health capital. The health capital is used to maintain the health of the labor force, and the physical capital is basic input for production activities. The product market clearing condition is:


(8) $$\begin{array}{l} y_{t}=c_{t}+v_{t}+e_{t} \end{array}$$

Where, *c*_*t*_ is consumption, physical capital and health capital investment are *v*_*t*_ and *e*_*t*_, respectively.

The law of motion equations for the physical capital and health capital are:


(9) $$\begin{array}{l} {\dot{k}}_{t}=v_{t}-\delta_{k}k_{t}-\left(b-d\right)k_{t} \end{array}$$

(10) $$\begin{array}{l} {\overset{˙}{h}}_{t}=e_{t}-\delta_{h}h_{t}-\left(b-d\right)h_{t} \end{array}$$

For the newly increased population in the economy with a proportion of (*b* − *d*), the corresponding investment is needed to form the initial natural endowment of physical capital and health capital. The family's investment in physical capital is *v*_*t*_, and the depreciation rate is δ_*k*_. The family's investment in health capital is *e*_*t*_, and the depreciation rate of health capital is δ_*h*_.

This paper introduces health capital, which can be understood as the sum of capital investment closely related to disease prevention, public medical infrastructure and residents' medical care. On the one hand, health capital can be used for the prevention and control of infectious diseases, reducing the contact rate between infected and uninfected population, and thereby inhibiting the spread of the virus; on the other hand, the health capital can also be used for the treatment of infectious diseases to increase the recovery rate of infected cases.

Based on Smith and McKenzie (31) and Smith et al. (32) we set the contact rate and recovery rate parameters to be time-varying and subject to government's public health policies. The parameters are defined as functions of health capital as follows:


(11) $$\begin{array}{l} \alpha =\alpha \left(h_{t}\right)=e^{-\xi h_{t}}\\ \;\gamma =\gamma \left(h_{t}\right)=1-e^{-\zeta h_{t}} \end{array}$$

Where, the contact rate is a monotonic decreasing function of health capital, and the recovery rate is a monotonic increasing function of the level of health capital. From a purely mathematical point of view, they are endogenous variables closely related to other endogenous variables. But in order to be consistent with the previous literature, we choose to treat them as time-varying parameters. Their connotations are just the same as the corresponding parameters in the canonical infectious disease dynamics model. The only difference is that they will change over time due to fluctuations in health capital. The parameters ξ and ζ represent the government's efforts in preventing and controlling the spread of the virus and improving the level of medical treatment, respectively. The former achieves control of the epidemic by increasing investment in prevention and control measures such as disease prevention and control of personnel contacts; the latter achieves control of the epidemic by increasing investment in patient diagnosis and treatment facilities and vaccine drugs and other treatment measures. The greater the prevention and control of the spread of the virus (the greater the ζ), the lower the contact rate (the smaller the α) will be; the greater the investment in the medical treatment system (the greater the ζ), the greater the recovery rate (the larger the γ) will be. Therefore, under different policy combinations, limited health capital will be distributed differently, which impose different effects on the control of the pandemic and macroeconomic dynamics.

### Utility Optimization

During the period of pandemic, the real labor supply in the economic system will be dynamically constrained by Equation (6), which in turn affects economic output and consumption. Therefore, compared with the canonical growth model, this paper emphasizes the influence of the pandemic on utility and production inputs. In order to explore the utility optimization problem of representative economic agents under the multiple constraints of economic and health conditions, this paper incorporates the variable of the proportion of uninfected population into the utility. The utility function is of the following form:


(12) $$\begin{array}{l} U_{t}\left(c_{t},l_{t}\right)=\ln\;\left(c_{t}\right)+\ln\;\left(l_{t}\right) \end{array}$$

Not only the consumption, the proportion of uninfected population (*l*_*t*_) will also change the utility. The larger the proportion, the higher the utility level. If the economic system is not affected by the pandemic, which means *l*_*t*_ = 1, then the utility function degenerates to the form widely used in the growth literature: *U*_*t*_(*c*_*t*_, *l*_*t*_) = ln (*c*_*t*_). Deviated from the literature, our set up makes it possible to depict the utility in both scenarios of pandemic shock and regular situation in one unified theoretical framework.

Different from the analysis of competitive equilibrium in the economy in the literature, this paper examines the equilibrium solution of the utility optimization problem from the perspective of the central planner. In central planner problem, labor in the economy will be affected by both the pandemic shock and government public policies. There will be endogenous interactions between labor force and health capital. For the central planner, the utility optimization problem can be written as:


(13) $$\begin{array}{l} \max\int_{0}^{\infty }e^{-\rho t}U_{t}\left(c_{t},l_{t}\right)dt \end{array}$$

where, the subjective discount rate is ρ, the corresponding constraints are Equations (6–11) and *k*_*t*_ ≥ 0, *h*_*t*_ ≥ 0, 0 < *l*_*t*_ ≤ 1.

The Lagrangian function is written as:


(14) $$\begin{array}{l} \ell_{t}=\ln(c_{t})+\ln(l_{t})+\lambda_{1,t}(y_{t}-c_{t}-e_{t}-\delta_{k}k_{t}-(b-d)k_{t})\\ +\lambda_{2,t}(e_{t}-\delta_{h}h_{t}-(b-d)h_{t})+\lambda_{3,t}(1-l_{t})(b+\gamma (h_{t})-\alpha \\ (h_{t})l_{t})+\lambda_{4,t}(1-l_{t})+\lambda_{5,t}h_{t} \end{array}$$

We solve for the first-order condition:


(15) $$\begin{array}{l} c_{t}:\lambda_{1,t}=1/c_{t} \end{array}$$

(16) $$\begin{array}{l} e_{t}:\lambda_{2,t}=\lambda_{1,t} \end{array}$$

(17) $$\begin{array}{l} k_{t}:{\dot{\lambda }}_{1,t}=-\lambda_{1,t}\left(\partial y_{t}/\partial k_{t}-\delta_{k}-\left(b-d\right)-\rho \right) \end{array}$$

(18) $$\begin{array}{l} h_{t}:{\dot{\lambda }}_{2,t}=\lambda_{2,t}\left(\delta_{h}+\left(b-d\right)+\rho \right)+\lambda_{3,t}\left(1-l_{t}\right)\\ \left(-\partial \gamma \left(h_{t}\right)/\partial h_{t}-l_{t}\partial \alpha \left(h_{t}\right)/\partial h_{t}\right)+\lambda_{5,t} \end{array}$$

(19) $$\begin{array}{l} l_{t}:{\dot{\lambda }}_{3,t}=1/l_{t}-\lambda_{1,t}\partial \gamma_{t}/\partial l_{t}+\lambda_{3,t}(b+\gamma (h_{t})\\ +\alpha (h_{t})(1-2l_{t})+\rho )-\lambda_{4,t} \end{array}$$

In addition, the following conditions must be met:


(20) $$\begin{array}{l} \lambda_{4,t}\geq 0,1-l_{t}\geq 0,\lambda_{4,t}\left(1-l_{t}\right)-0,\lambda_{5,t}\geq 0,h_{t}\geq 0,\\ \lambda_{5,t}h_{t}=0 \end{array}$$

Also, the following three transversality conditions should be met:$\underset{t\to \infty }{\text{lim}}e^{-\rho t}\lambda_{1,t}k_{t}=0,\underset{t\to \infty }{\text{lim}}e^{-\rho t}\lambda_{2,t}h_{t}=0,\underset{t\to \infty }{\text{lim}}e^{-\rho t}\lambda_{3,t}l_{t}=0$.

### Equilibria

Based on the above conditions, it is obvious that the model has multiple equilibria. We start to analyze economic equilibrium from the dynamic Equation (6). When the equilibrium is achieved, all endogenous variables, both pandemic and economic ones, reach their steady-state values. And the market clears. Therefore, we let *i*_*t*_ = 0 to get:


(21) $$\begin{array}{l} \left(1-l_{t}\right)\left(b+\gamma -\alpha l_{t}\right)=0 \end{array}$$

It means that the labor supply reaches a steady-state value and its growth rate is 0. It also means that under the government's intervention against the pandemic, the proportion of uninfected population in the economy also remains unchanged. It is easy to find there are two different equilibria, corresponding to *l*_*t*_ = 1 and *l*_*t*_ = (*b* + γ)/α, respectively. The former equilibrium means that there is no spread of virus, and the proportion of population get infected in the economy is zero. This equilibrium state is similar to that of the classic growth model that does not subject to infectious disease. The latter equilibrium represents that although infectious diseases have not been completely eliminated, their marginal impact on labor supply has been offset by intervention measures and the accumulation of healthy capital, and the proportion of healthy people in the total population is in a stable state.

For economic variables *x*, we denote $\overset{-}{x}$ as the steady-state value in the equilibrium without any infectious diseases, and $\tilde{x}$ as the steady-state value in the equilibrium subject to a pandemic. Thus, there are two propositions can be obtained.

#### Proposition 1

When the economic system is not subjected to infectious disease, equilibrium values of labor, and health capital satisfy the following equations:


(22) $$\begin{array}{l} \overset{-}{l}=1,\overset{-}{h}=0 \end{array}$$

And the steady state capital will be:


(23) $$\begin{array}{l} \overset{-}{k}={\left(\frac{\delta_{k}+\beta +b-d}{\eta a}\right)}^{\frac{1}{\eta -1}} \end{array}$$

#### Proof

When the economic system is not subjected to infectious disease, the whole population can provide labor, so $\overset{-}{l}=1$. From Equations (15, 16, 18), we know that in the equilibrium, λ_5_ > 0. Because the condition $\lambda_{5}\overset{-}{h}=0$ holds, it is obvious that $\overset{-}{h}=0$. Since Equation (17) equals 0, the steady state capital can be obtained by combining Equation (7,17).

It can be found that in the absence of infectious diseases, the steady state of the model is quite similar to that of canonical growth model. But the introduction of infectious disease transmission changes the results significantly. First, there are dynamic interactions among labor supply, health capital, and disease transmission; second, due to the investment in health capital, the optimal state of resource allocation deviates from the optimal steady state. Finally, the steady-state values of output and consumption have also changed due to the impact of infectious diseases. The following proposition defines the steady-state value of economic variables in the presence of infectious diseases.

#### Proposition 2

When there is an infectious disease, only uninfected population can provide labor. If the economy is in equilibrium, the labor supply satisfies $\tilde{l}-\left(b+\gamma \left(\tilde{h}\right)\right)/\alpha \left(\tilde{h}\right)$, and health capital satisfies $0<\left(b+\gamma \left(\tilde{h}\right)\right)/\alpha \left(\tilde{h}\right)<1$. The steady-state values of capital and labor are determined by the following equations:


(24) $$\begin{array}{l} \tilde{k}={\left(\frac{\delta_{k}+\rho +b-d}{a\eta {\tilde{l}}^{1-\eta }}\right)}^{\frac{1}{\eta -1}} \end{array}$$

(25) $$\begin{array}{l} \tilde{l}=(-(\frac{\left(\alpha (\tilde{h})+\rho -\gamma (\tilde{h})-b)(\delta_{h}+\rho +b-d\right)}{\left(1-\tilde{l})(\alpha^{\prime }(\tilde{h})\tilde{l}-\gamma \alpha^{\prime }(\tilde{h})\right)}\\ {+\frac{\tilde{c}}{\tilde{l}})/a(1-\eta ){\tilde{k}}^{\eta })}^{\frac{1}{\eta }} \end{array}$$

#### Proof

When the economic system is subjected to infectious disease, only uninfected people provide labor. The labor supply should satisfy $\tilde{l}\neq 1$. According to Equation (21), the labor supply should be $\tilde{l}=\left(b+\gamma \left(\tilde{h}\right)\right)/\alpha \left(\tilde{h}\right)$, and the health capital is defined by the inverse function of health capital as $\tilde{h}={\tilde{l}}^{-1}\left(\tilde{h}\right)$. At the same time, since $0<\tilde{l}<1$ holds, the inequality $0<\left(b+\gamma \left(\tilde{h}\right)\right)/\alpha \left(\tilde{h}\right)<1$. Similar to the proof for Proposition 1, the steady-state value of capital can be obtained as Equation (24). In the equilibrium, Equations (18,19) are equal to 0, combined with Equations (15,16), the steady state value of labor can be obtained as shown in Equation (25).

## Calibration and Steady State Analysis

### Parameter Calibration

In order to further analyze the impact of pandemic on economic equilibrium, this paper calibrates the model to the Chinese economy and solve the steady-state values of macroeconomic variables. Based on the birth rate and death rate reported by National Bureau of Statistics of China from 2009 to 2019^4^, we set the birth rate (*b*) in the model to 1.2% and the death rate (*d*) to 0.7%. Based on the average Shibor interest rate in China from 1999 to 2019^5^, we set the discount rate (ρ) to 2.4%. In the production function, the productivity (*a*) is standardized to 1, and the capital share (η) is calibrated to be 0.33. We set the physical capital depreciation rate (δ_*k*_) and the health capital depreciation rate (δ_*h*_) to 5%. In the model, government's pandemic prevention and control efforts are captured by the parameter ξ in Equation (11), which is set to be 1.0. At the same time, the government's initiative to invest in medical treatment are captured by the parameter ζ which is also calibrated to be 1.0. The connotations and values of model parameters are summarized in Table 1.

**Table 1:** Model parameter calibration.

**Parameter**	**Connotation**	**Value**
*b*	Birth rate	1.2%
*d*	Population mortality rate	0.7%
*p*	Discount rate	2.4%
*a*	Firm productivity	1.0
η	Capital share in production function	0.33
δ_*k*_	Depreciation rate of productive capital	5%
δ_*h*_	Health capital depreciation rate	5%
ξ	The government's efforts to prevent and control the spread of the virus	1.0
ζ	The government's initiative to investment in medical treatment	1.0

### Steady State Analysis

**Figure 2:**
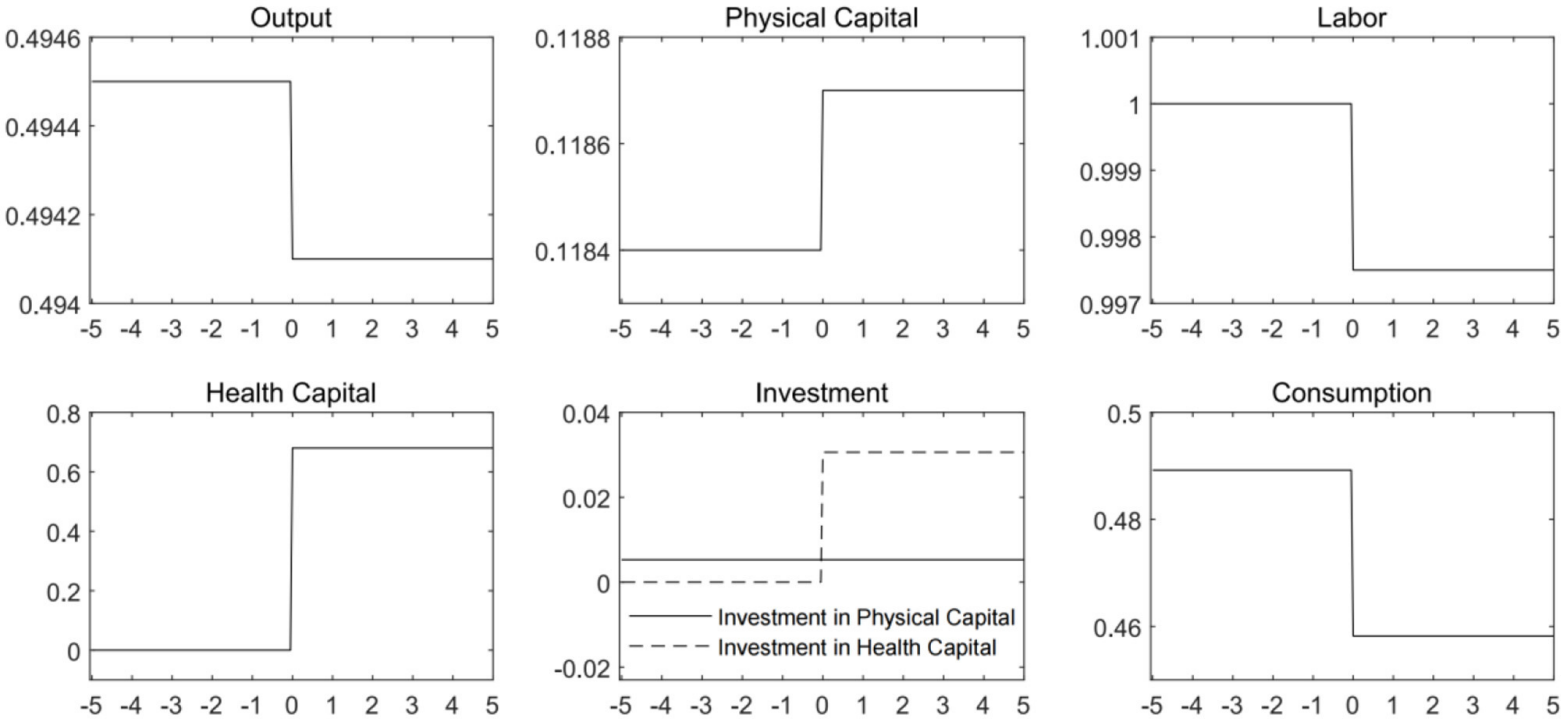
Economic steady states and pandemic shock. It is assumed that the pandemic shock hit the economy at time *t* = 0. Before that, the economy is not subjected to any infectious disease.

Based on the calibrated model, we solve for the economic equilibrium before and after the outbreak of infectious disease. We assume that after the pandemic shock, the economy reaches the new equilibrium instantly. Two steady states are presented in Figure 2 for comparative analysis. The horizontal axis is time, and we assume that the time of outbreak is *t* = 0. Before the outbreak, the entire population in the economy was able to provide labor to manufacturers. After the outbreak, labor supply will be restricted by the spread of infectious diseases.

Before the outbreak of infectious disease, there is no need to accumulate health capital. After the outbreak, some individuals are unable to work due to illness or the pandemic prevention and control measures. After the pandemic hitting economy, the equilibrium labor supply declines. In order to contain the spread of the virus, economic agents need to accumulate health capital. The investment in health capital increases from 0 to a positive level, and the stock of health capital rises since the outbreak. Due to the decrease in labor supply, physical capital investment rose slightly, but the increase is much smaller than that of health capital investment. The increase in physical capital cannot completely offset the negative impact on output caused by the reduction in labor supply. The steady-state value of aggregate output drops below the level before the pandemic outbreak. Along with the decrease in output and the increase in investment, consumption has dived significantly. These results are consistent with previous literatures such as Chakraborty et al. (14) and Goenka and Liu (20).

The above analysis justifies that outbreak of pandemic will directly lead to a decline in aggregate demand and output in the economy. At the same time, fighting the epidemic requires the accumulation of additional health capital. Healthy capital investment consumes part of the economic output. And the production of healthy capital will squeeze out the factor inputs required for the production of other products. The more investment flows into the health sector, the more effective the prevention and control of infectious diseases will be. As a result, there would be higher recovery rate for the infected population. The optimal health capital level is endogenously related to the dynamics of spread of the virus. When formulating relevant pandemic prevention and control policies, the government must consider the combination of different types of measures targeting the contact rate and recovery rate, as well as the trade-off between disease prevention and control and economic development.

## Policy Analysis

### Public Health Policy

To contain spread of virus, the government should resort to serious measures. The most direct responses from the Chinese government are social distancing, mask wearing and quarantine the infected cases. After that, the government provides vigorously support for the research and development of vaccines and treatment methods. By doing so, it is able to improve the recovery rate of patients, and reduce the scale of infection.

The research conducted by Zhong Nanshan's team fully demonstrated the important role of the government's swift and effective prevention and control measures against the spread of the virus in curbing the pandemic (23). And our recognition of the positive impact of health capital accumulation in fighting the pandemic is quite consistent with them.

Besides that, we have considered the possibility that limitation of resources leads to the non-linear benefits of the accumulation of health capital, and also that excessive accumulation of health capital may bring undesirable consequences. The steady-state of health capital represents the optimal value under the dual goals of curbing the pandemic and ensuring economic growth. The changes in the government's public health policies will not only directly lead to changes in pandemic related parameters, but also affect health capital and other economic variables through the dynamic interaction between the spread of virus and health capital. In order to further discuss the choice of government's public health policy, we have solved for the steady-state values of economic variables with different parameters portrait the pandemic prevention and control efforts and medical treatment investment.

**Figure 3:**
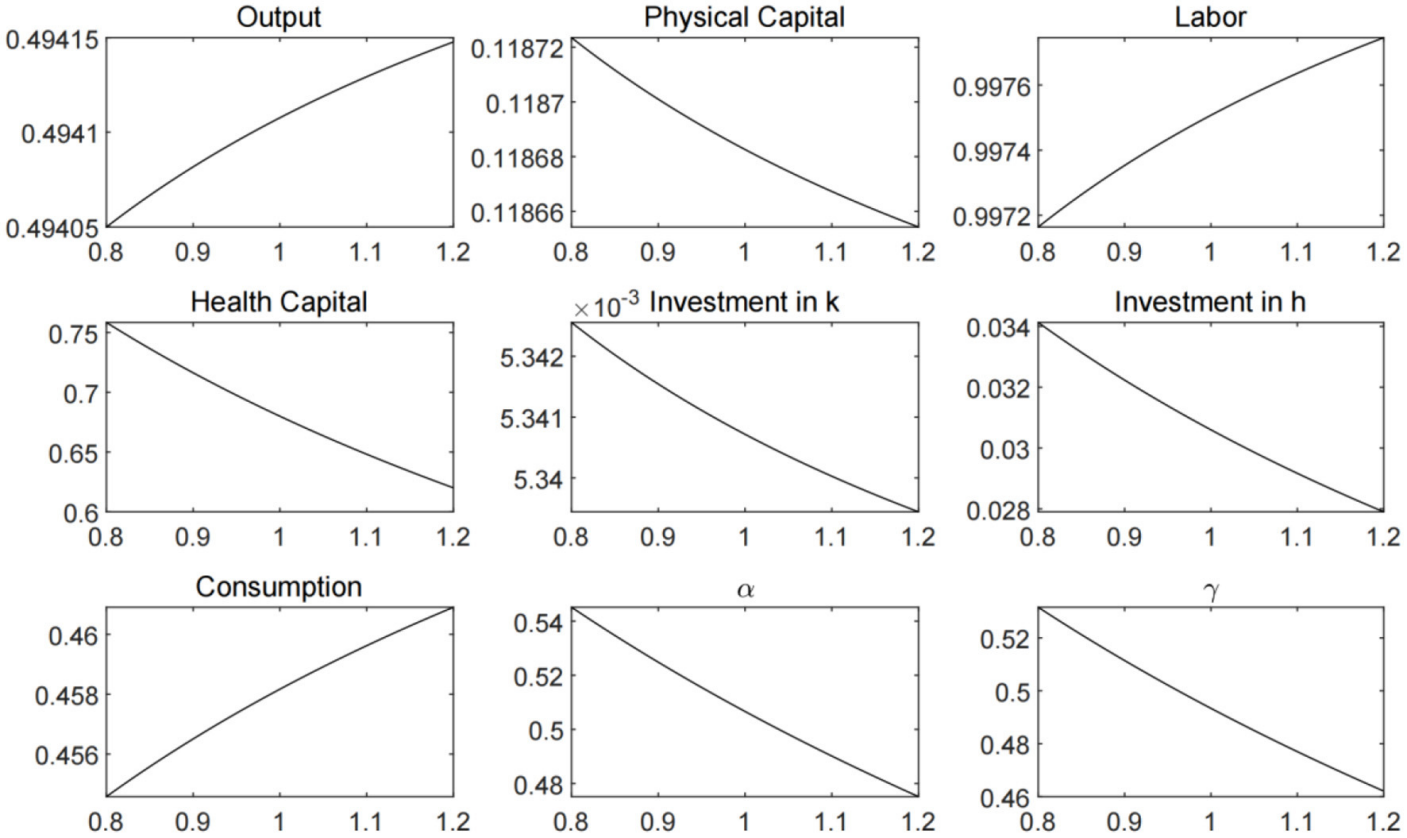
Economic steady states under different ξ values.

In Figure 3, we plot the economic steady states that solved with different ξ values, which represent varied pandemic prevention and control efforts. From Equation (11), it can be inferred that the greater the government's efforts to prevent and control the spread of the virus, the lower the contact rate and the demand for health capital will be. Given that the proportion of the uninfected population in the total population follows the path of change described by the differential Equation (6), the decline in the contact rate can increase the supply of labor, and at the same time, the substitution effect of labor on capital causes a decrease in the level of physical capital investment. It can be seen from the figure that the comprehensive effect of changes in labor and capital on the production of firms is to promote the increase in output, while the consumption level rises accordingly. However, it is worth noting that under the condition that the intensity of medical treatment remains unchanged, the decline in health capital causes a decline in the recovery rate, which has a negative impact on labor supply and economic output.

**Figure 4:**
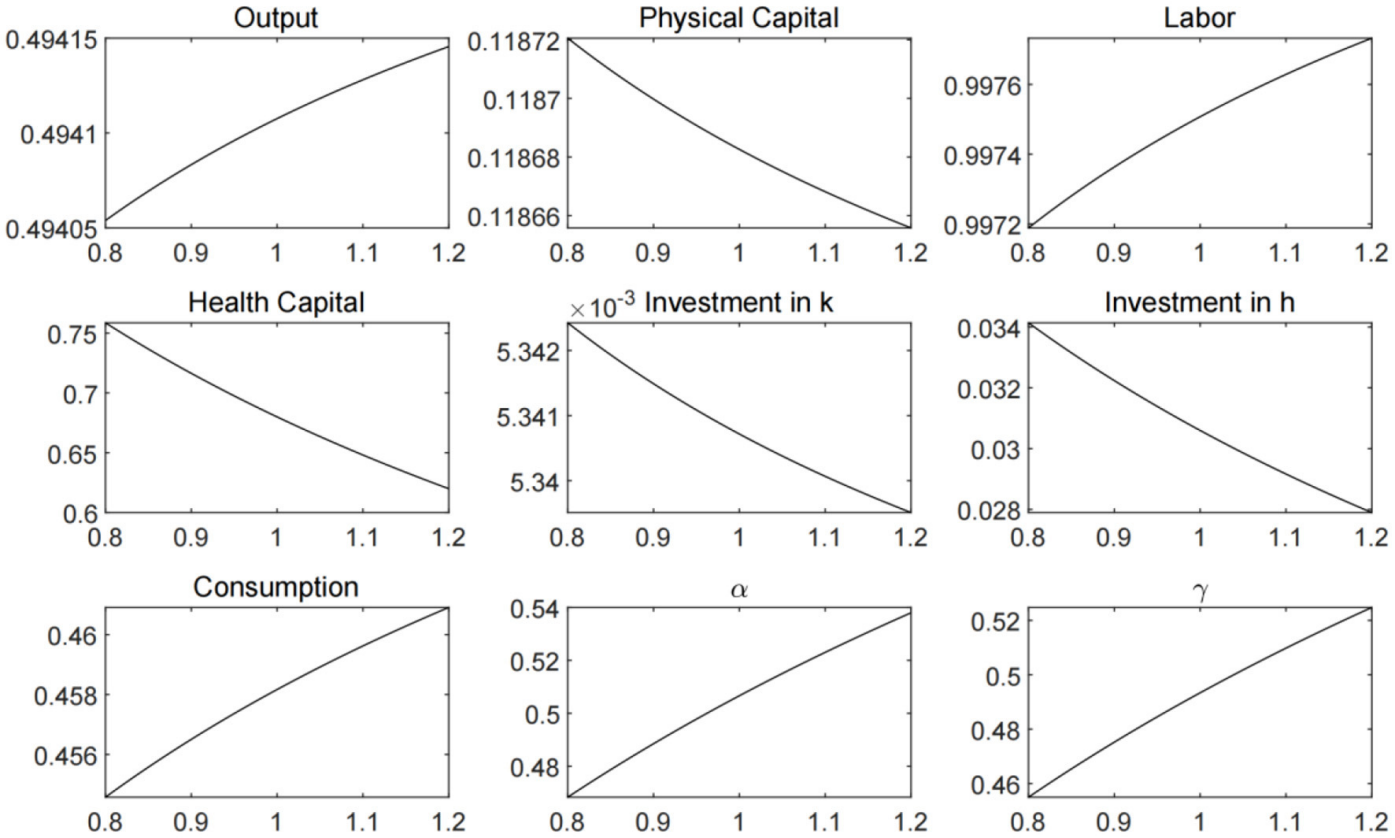
Economic steady states under different ζ values.

In Figure 4, we plot the economic steady states that solved with different ζ values, which represent government's initiative to investment in medical treatment. The increase in medical treatment investment can bring about the growth of labor supply, output and consumption. However, health capital investment will fall, triggering an increase in contact rates, and also have a negative impact on labor supply and economic output. Therefore, we need to further analyze the different combinations of public health policy parameters.

**Figure 5:**
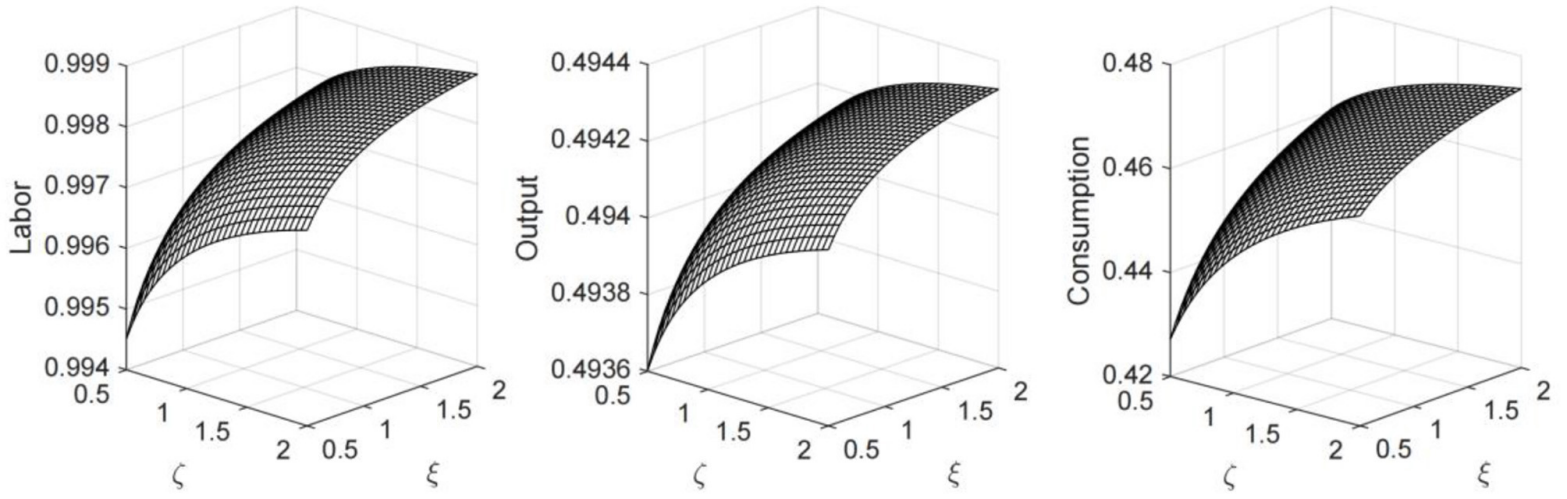
Economic steady states under different combination of ξ and ζ values.

In order to better analyze the effect of the government's public health policy, we set different values for the parameters ξ and ζ, and plot the steady states of labor, output, and consumption in Figure 5. The results suggest that the strengthening of public health policies can increase labor supply in the economy and promote output and consumption. However, the marginal effect of public health policy is diminishing. When the parameters reach the level above 1.5, the marginal increment of output and consumption becomes extremely small. Moreover, for pandemic prevention and control measures or medical treatment investment policy alone, the effect on economic development is limited. Only when the two directions of public health policies are combined can we achieve the best results.

### Economic Policy

The outbreak of the COVID-19 imposes severe constraints on labor supply and total demand. China has experienced economic austerity and predicament. The previous steady state analysis shows that the implementation of public health policies can lift the steady state of economic variables such as output and consumption to a certain extent. But further economic recovery requires more support and macroeconomic stimulus. When the spread of virus is gradually under control, the resumption of production and restore of social order become the priority for the government. However, the normalization of economic activity is accompanied by more frequent interpersonal interaction and contact, which may lead to higher probability of an outbreak. So, it is absolutely necessary to pay attention to the counterproductive effect of economic stimulus policy for the policy makers.

#### Resumption of Production

The China Enterprise Confederation established a survey on the resumption of work and production of China's top 500 manufacturing companies.^6^ The survey shows that during the survey period from February 18 to 20, 97.08% of surveyed companies have resumed production, the average employee attendance rate was 66.17%, the average operating rate of member enterprises was 75.24%, and the average capacity utilization rate was 58.98%. The experiences from the firms that have been back to business imply that resuming work and production under the premise of doing a good job in epidemic prevention and control can directly increase capacity utilization and bring about an increase in productivity.

**Figure 6:**
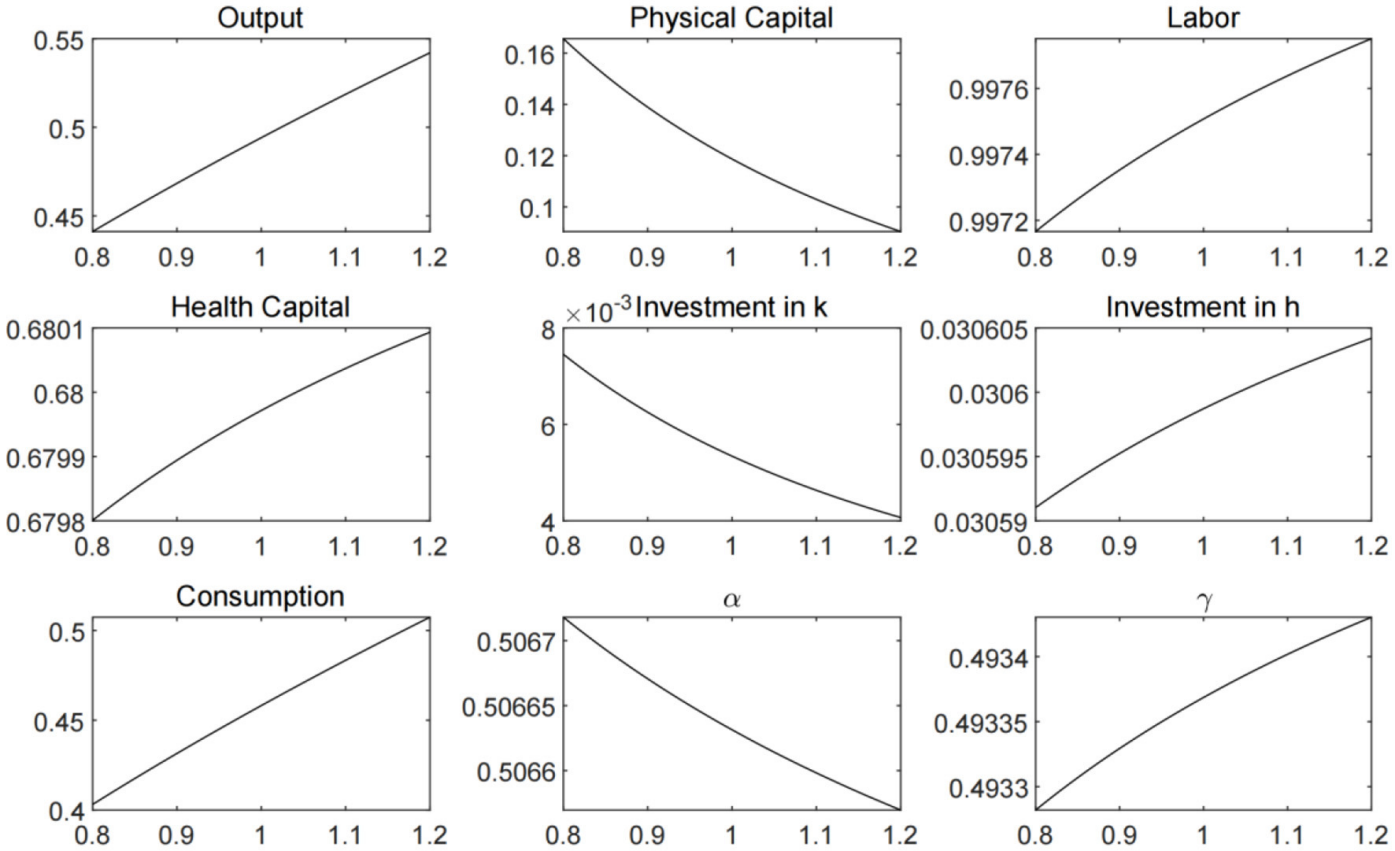
Economic steady state under different productivity levels.

We are not able to directly investigate the epidemiological and economic impact of the resumption of firm production based on our model. But it can be inferred from the change of firm productivity and its consequences. The degree of enterprise's resumption of production is highly positively correlated with its productivity. We set different values for the productivity parameters to approximate the degree of resumption. And then, the economic steady states are calculated plotted in Figure 6. There is a very intuitive conclusion that incremental productivity boost output and consumption. But the impact of productivity increase on labor is two-fold. On the one hand, the increase in productivity will reduce the demand for capital and labor by manufacturers. On the other hand, higher productivity level leads to output growth, which is beneficial for the health capital accumulation. The increase in health capital can reduce the infection and increase the recovery rate, resulting in better pandemic prevention and higher level of labor supply.

The above analysis suggests that as long as the resumption of production is conducted under the premise of splendid prevention and control measures, not only can it effectively stimulate economic recovery, but also help the accumulation of health capital and resulting in significant inhibitory effect on the pandemic. At the same time, timely and effective pandemic prevention and control are inseparable from the use of health capital. Overly strict prevention measures may cause delays in resuming work and production, and impose a negative impact on the accumulation of health capital. Therefore, it is necessary to carefully weigh the prevention costs brought by the resumption of work and production and the economic output.

### Economic Stimulus Policy

After resumption of production, the economic activities are brought back gradually. In order to achieve economic recovery, most countries in the world have proposed economic stimulus plans. From the historical experience, infrastructure investment is one of the most direct and effective means to boost the economy, especially in the context of relatively insufficient domestic demand following the pandemic shock. Compared with stimulating consumption, increased investment is more likely to help the economy to recover effectively in the short term.

It's worth noting that different direction of infrastructure investment imposes differentiating impact on labor. There exists significant substitution of technology for labor in certain industries. Investing in such industries may actually lead to a decline in the labor demand that is already not optimistic after the pandemic shock. China is also facing downward pressure on the economic growth caused by deleveraging and trade frictions in the post pandemic period. In order to avoid extensive investment in traditional infrastructure investment, the Chinese government choose to identify a number of high-tech industries, like AI and 5G, as the destination of “new infrastructure” stimulus package^7^.

**Figure 7:**
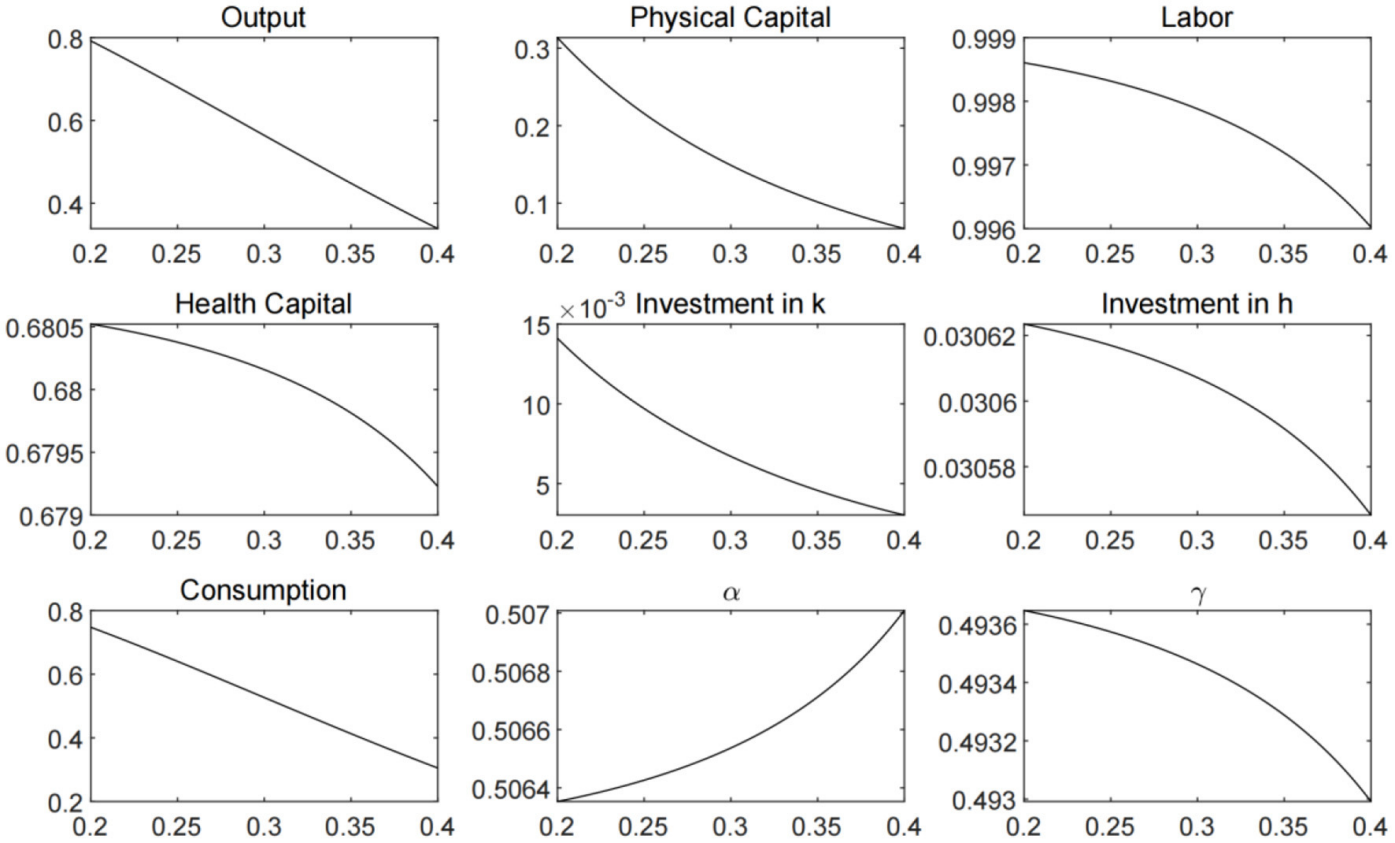
Economic steady state under different capital shares.

The logic that “new infrastructure” investment can promote output by increasing the scale of investment in the economy is intuitive. However, we also need to be aware of the potential “labor substitution” effect of this type of infrastructure investment. Schumpeter (33) pointed out that technological innovation will reduce the demand for labor in the production process and cause unemployment. As advanced forms of automation technology, high-tech applications such as artificial intelligence have the ability to replace part of mental labor, which may impose a more significant substitution effect on labor (34). As mentioned above, most of the investment in the “new infrastructure” goes to the high-tech fields. There is a complementary relationship between the capital leaned technological progress and capital investment, which will naturally impose substitution effect on labor. In order to explore the potential impact of increasing investment in capital-leaned high-tech industries on labor and output, we solve the steady state under different capital share and present the results in Figure 7. It can be seen that when capital share rises, other macroeconomic variables decline, including labor, output, and consumption. More importantly, the increase in the share of capital will also crowd out the investment of health capital, which will further push up the contact rate and reduce the recovery rate.

Based on the above discussion, the government needs to carefully assess the potential impact on employment and output when increasing investment in high-tech industries. Optimal stimulus policies should consider the construction of both capital-intensive and labor-intensive projects, and weigh multiple policy goals such as stimulating output and ensuring employment. In this way, the excessive substitution of labor by technological progress and unemployment can be avoid.

## Conclusion

This article combines economic theory with infectious disease models, and analyzes the long-term impact of the novel coronavirus pandemic on labor supply, household consumption and economic growth. And the simulation shed light on public health policies and economic stimulus policies under different conditions Implementation Effect.

The results show that the outbreak of COVID-19 pandemic directly affect labor supply and output, resulting in a significant negative impact on the economic growth. The accumulation of health capital can inhibit the spread of infectious diseases and improve the recovery rate. Thus, the pandemic prevention and control become more promising and ultimately lead to the growth of economic output and consumption. Public health policies can enhance the significant role of health capital in promoting pandemic prevention and economic growth, but the effects are marginal diminishing. So, overinvestment in public health should be avoided. And, only when the government increases investment in the prevention measures and medical treatment at the same time, the negative consequences of pandemic are able to be minimized. Resuming production and work is not only conducive to economic recovery, but also imposes a positive effect on pandemic prevention through the accumulation of health capital. Investing infrastructure is definitely an effective way to boost the economy. However, “new infrastructure” investment in high-tech industries may otherwise impose substitution effect on labor, which could push up unemployment rate. It is clear that only on the premise of effectively dealing with the spread of the epidemic, economic development is a viable option.

As the COVID-19 pandemic has repeatedly erupted around the world, China's experience has its unique value. In the early stage of the outbreak, strict quarantine measures, tracking of close contacts, and the control of social distancing had a considerable negative impact on the Chinese economy. However, the advantage is that the severe measures have created prerequisite for the rapid restoration of the social and economic order. The Chinese economy has begun a strong rebound in the second quarter after the outbreak of the epidemic.

The theoretical model constructed in this article is a highly abstract macroeconomic system under the pandemic. So, it has its limitations. This article assumes that all participants in the economic system are fully rational. The impact of the epidemic on economic agents' expectations and preventive savings are not considered in the model. And the model is not able to characterize the positive effect of vaccination on suppressing the spread of the virus. In future research, the authors hope to further correlate the dynamics of virus transmission with vaccines, economic man expectations, and financial activities within a dynamic general equilibrium framework.

## Data Availability Statement

Publicly available datasets were analyzed in this study. This data can be found here: http://www.stats.gov.cn/.

## Author Contributions

LX: conceptualization, validation, writing original draft, and supervision. MT: methodology, writing—review and editing, and visualization. ZY: writing—review and editing, project administration, and funding acquisition. MZ: software, resources, and data curation. SL: formal analysis and investigation. All authors contributed to the article and approved the submitted version.

## Funding

We acknowledge the financial support from Shandong Provincial Natural Science Foundation (Grant Number: ZR2020QG032), Shandong Provincial Social Science Planning Office (Grant Numbers: 21DGLJ12; 21DJJJ02), Taishan Scholars Program of Shandong Province, China (Grant Numbers: ts201712059; tsqn201909135), and Youth Innovative Talent Technology Program of Shandong Province, China (Grant Number: 2019RWE004). All errors remain our own.

## Conflict of Interest

The authors declare that the research was conducted in the absence of any commercial or financial relationships that could be construed as a potential conflict of interest.

## Publisher's Note

All claims expressed in this article are solely those of the authors and do not necessarily represent those of their affiliated organizations, or those of the publisher, the editors and the reviewers. Any product that may be evaluated in this article, or claim that may be made by its manufacturer, is not guaranteed or endorsed by the publisher.

## Footnotes

^1^On January 31, 2020, the National Health Commission of China stated at a press conference that patients who have recovered from the new coronavirus pneumonia are subject to reinfection. On October 27, 2020, the CDC of USA confirmed cases of reinfection.

^2^This paper chooses the more intuitive term “uninfected population” when discussing relevant economic scenarios. In the following text, we will use “susceptible people” and “uninfected people” interchangeably.

^3^See Gersovitz and Hammer (30) for a detailed discussion.

^4^Data source: official website of the National Bureau of Statistics of China.

^5^Shibor stands for the Shanghai inter-bank offering rate.

^6^Survey report available at: https://www.acfic.org.cn/zt_home/zcmq2020/zcmq2020_dt/202002/t20200226_158162.html.

^7^The high-tech industries include: (1) 5G base station construction; (2) UHV; (3) Intercity high-speed railway and urban rail transit; (4) New energy vehicle charging pile; (5) Big data; (6) Artificial intelligence; (7) Industrial Internet.
